# Tyrosine bioconjugation – an emergent alternative

**DOI:** 10.1039/d0ob01912g

**Published:** 2020-10-28

**Authors:** Peter A. Szijj, Kristina A. Kostadinova, Richard J. Spears, Vijay Chudasama

**Affiliations:** a Department of Chemistry , University College London , London , UK . Email: v.chudasama@ucl.ac.uk

## Abstract

Protein bioconjugation is an increasingly important field of research, with wide-ranging applications in areas such as therapeutics and biomaterials. Traditional cysteine and lysine bioconjugation strategies are widely used and have been extensively researched, but in some cases they are not appropriate and alternatives are needed or they are not compatible with one another to enable the formation of dually (and distinctly) modified dual-conjugates (an increasingly desired class of bioconjugates). Here we review the heretofore less explored approach of tyrosine bioconjugation, which is rapidly becoming a constructive alternative/complement to the more well-established strategies. Herein we present an overview of the field, and then focus on promising recent methods that can achieve high conversion and chemoselectivity. This suggests that not only can tyrosine bioconjugation be used in conjunction with cysteine and lysine modification to obtain proteins with multiple different modifications, it is also becoming a stand-alone alternative to these more traditional methods.

## Abstract 2

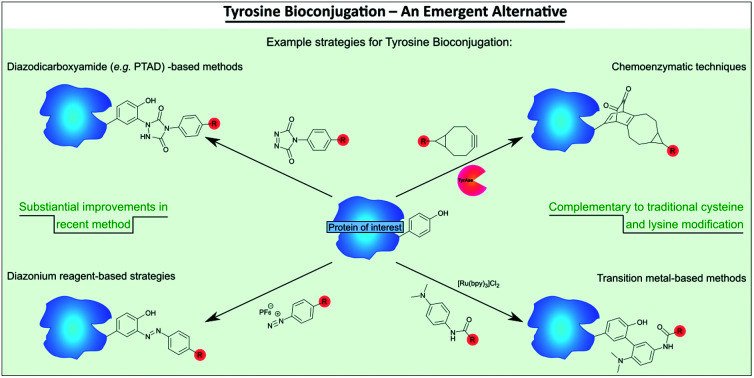
A review of the heretofore less explored approach of tyrosine bioconjugation, which is rapidly becoming a constructive alternative/complement to the more well-established strategies, is provided.

## Introduction

Protein bioconjugation is a rapidly progressing field of research owing to the wide variety of applications these techniques provide.^1^ These include powerful tools for the investigation of biological systems by improving methods (*e.g.* super-resolution microscopy, flow cytometry) and acting as fluorescent biosensors to detect the distribution of molecules of interest in live cells.^2^ Another application is in the development of well-defined biomaterials. Especially versatile are modified viral capsids, *e.g.* tobacco mosaic virus (TMV) capsid monomers with attached fluorophores that can self-assemble to produce systems for synthetic light harvesting.^3^,^4^

Perhaps, one of the areas receiving the most attention is the use of protein bioconjugates as therapeutics.^5^,^6^ The attachment of polyethylene glycol (PEGylation) has been long known to improve the pharmacokinetic properties of biologics by increasing their bioavailability and reducing immunogenicity. As a result, many PEGylated proteins have been approved for treatment (*e.g.* PEG-interferons, pegvisomant, pegaspargase).^6^ Another important group of therapeutics, especially in cancer therapy, are antibody–drug conjugates (ADCs).^5^ These constructs combine the cytotoxicity of small-molecule drugs with the specificity of monoclonal antibodies, therefore achieving targeted delivery and reducing off-site effects and the resulting toxicity. Currently, eight ADCs are approved for use (*e.g.* brentuximab vedotin and trastuzumab emtansine), with many more in clinical trials.^7^

A wide variety of methods have to date been developed for chemical protein modification.^1^ Some traits have been found to be generally desirable for these approaches to be considered promising. For instance, mild reaction conditions are necessary in order to avoid denaturation and thus preserve protein function. As such, these reactions are ideally carried out in aqueous media, at room temperature and at neutral pH. In addition, homogeneity of the product is often important, especially for bioconjugates with medicinal application, because a mixture of species with different drug-loading has narrower therapeutic window and variable pharmacokinetic properties.^5^ Thus modification methods should chemoselectively target a specific functionality (usually an amino acid side-chain) and ideally be site-selective, *i.e.* target only a certain specific amino acid residue or a set of residues. Therefore, significant effort is focused on the development of site-selective methods for protein modification that target a specific amino acid residue and consequently yield a homogenous product.

Traditional methods for bioconjugation include lysine and cysteine modification, which are very thoroughly researched, well optimised and widely applicable.^1^ In comparison, the field of tyrosine modification is far less developed.^8^ However, tyrosine has some advantageous properties for bioconjugation approaches, for example, due to its amphiphilic nature, it is relatively rarely surface-exposed; this makes it a good target for site-selective modification. By contrast, lysine is much more abundant and as a result, site-selectivity is very difficult to achieve as demonstrated with the ADCs Kadcyla and Mylotarg.^5^ Cysteine modification yields less heterogeneous products, however, the thiosuccinimide linkage produced after conjugation with commonly used maleimide reagents can undergo retro-Michael deconjugation and is thus unstable;^9^ although it is appreciated that advances to combat this have been made.^1^,^9^ Moreover, the high reactivity of cysteine may lead to its oxidation or protein dimerization *via* disulfide bond formation, if introduced on the protein surface, issues which do not apply in the case of less reactive tyrosine residues. Additionally, cysteines often require reduction prior to conjugation (either as they are capped by thiols in production, exist as protein dimers or as they need to be liberated from disulfide bonds in the native protein structure), while tyrosine residues are in their reactive states by default.

Novel methods for tyrosine modification would complement the existing cysteine and lysine conjugation strategies, especially if these are orthogonal; allowing the attachment of different cargoes on the same protein.^10^ More fundamentally, in some cases the option of cysteine and/or lysine modification may not be applicable to certain proteins and an alternative amino acid modification strategy is essential. This review aims to summarise the most important existing methods for tyrosine bioconjugation and to inspire future research in the field. In the preparation of this manuscript, a complementary review on tyrosine modification was published and we wanted to explicitly acknowledge the excellent reviewing in the area by Gouin and co-workers.^11^

## Chemical approaches for tyrosine modification

### Mannich-type reactions

**Scheme 1:**
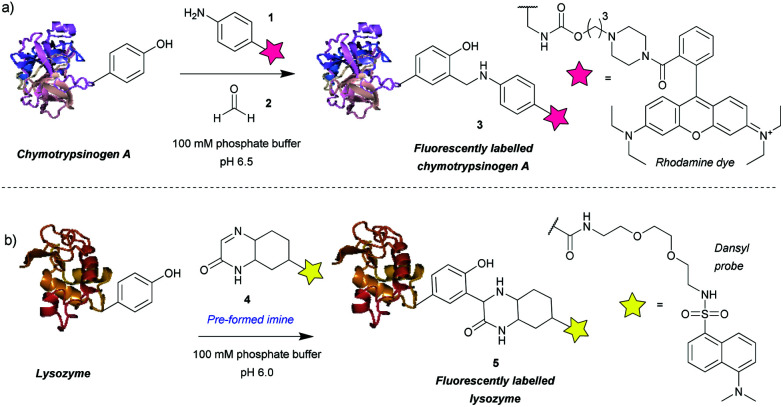
Tyrosine bioconjugation *via* Mannich-type reactions. (a) Tri-component Mannich-type reaction. (b) Tyrosine-modification with a pre-formed imine.

Among the first methods developed for tyrosine modification were those relying on Mannich-type reactions (Scheme 1). In this context the three-component Mannich-type reaction involved *in situ* formation of an imine from a substituted aniline **1** and an aldehyde **2**, which subsequently reacted with a tyrosine residue, resulting in carbon–carbon bond formation to yield bioconjugate **3** (Scheme 1a). The reaction was used for the attachment of fluorophores^12^ and synthetic peptides^13^ to chymotrypsinogen. An advantage of this method is the theoretical ability to add two functional groups concurrently. However, as the reaction proceeds best with formaldehyde, which is thus the most frequently used reagent, in practice only one functionality is usually introduced. Some aldehydes, *e.g.* crotonaldehyde and 2-furaldehyde, were shown to be less reactive as evidenced from the less intense fluorescent bands compared to Coomassie staining on SDS-PAGE, and others (glyoxylic acid, benzaldehyde, propionaldehyde) did not yield modified protein at all. The choice of aniline is also limited, *i.e.* to those bearing electron donating groups (alkyl, methoxy), as those with strongly electron withdrawing groups (*e.g.* nitro, carboxylic) afforded little or no conversion. Moreover, although the reaction conditions are mild (aqueous buffer pH 6.5, r.t. to 37 °C), long reaction times are needed (18 h to attach rhodamine, 24 h for peptides) and the efficiency is moderate, *e.g.* 66% labeling with rhodamine. In addition, the reaction often leads to a mixture of singly and doubly modified products.

The main issue with this method is the low selectivity for tyrosine. An NMR study on the modified proteins, detected products of nucleophilic attack on the imine by tryptophan and cysteine residues.^14^ The number and site of the side reactions depended on the protein in question, for example, chymotrypsinogen is modified only on tyrosine, whereas in the case of lysozyme only 40% of the modifications occurred on tyrosine, with the other 60% being on tryptophan. This poses a limitation on the use of the three-component Mannich-type reaction for tyrosine selective protein modification and highlights the potential benefits of improving the chemoselectivity of the method.

An attempt was made to address these issues by reaction preformed cyclic imine **4**. The authors reasoned that these molecules would re-form the imine after hydrolysis – so reaction could work at higher pH and that these compounds should not isomerize to enamines, preventing dimerization that would otherwise lower the imine concentration.^15^ Initial model studies showed that the cyclic imine did not react with 3-methylindole, thus suggesting a lack of reactivity towards tryptophan, however, the imine did undergo addition to 1-dodecanethiol (thus indicating it could undergo possible side reactions with cysteine).^16^ A set of proteins, lysozyme from chicken egg white, α-chymotrypsinogen A type II from bovine pancreas, myoglobin from equine skeletal muscle, carbonic anhydrase isozyme II from bovine erythrocytes, and cytochrome C from horse heart, were used with cyclic imine **4** to form conjugate **5**. Unfortunately, even after a reaction time of 4 days, the method only modified one protein (lysozyme) to a significant degree out of the five proteins tested (Scheme 1b).^16^ It is worth noting that chymotrypsinogen probably self-digested under the conditions leading to no observable modification. Gratifyingly, lysozyme was shown to afford the monoadduct by MS analysis, and thus the authors reasoned that as compound **4** was shown to be tyrosine-selective in model studies^15^ (apart from reacting with thiols), the conjugation was probably site-selective.

### Diazonium reagents

**Scheme 2:**
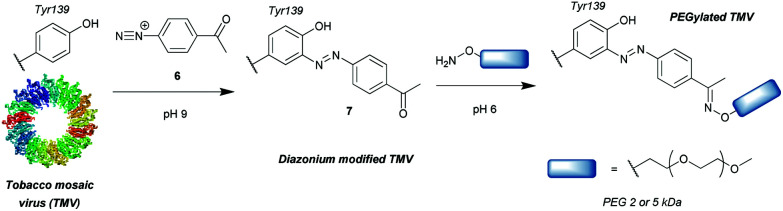
General strategy for tyrosine bioconjugation with diazonium reagents.

Another group of compounds used to modify tyrosine residues are diazonium reagents **6**, which were used to modify tyrosine residues on the MS2 bacteriophage^17^ and tobacco mosaic virus^18^ capsids (Scheme 2) to form conjugates in the form of **7**. Excellent conversions of more than 90% were achieved over short reaction times between 15 min and 2 h (pH 9, 4 °C), with diazonium compounds having electron-withdrawing groups at the *para*-position. The nitro-substituted compounds worked particularly well, however these reagents have limited application in further reactions and three additional steps were needed before the final hetero-Diels–Alder conjugation allowed installation of a functional moiety. Schlick *et al.* instead developed a ketone-containing diazonium salt, which allowed for subsequent conjugation of PEGs (*M*_w_ = 2000 and 5000) *via* oxime formation. In addition, both capsids were modified site-selectively, perhaps due to the good solvent accessibility of only one tyrosine residue per protein monomer, proving that depending on the particular protein, tyrosine modification can be a very powerful strategy for bioconjugation.

Whilst successful for viral capsid modification, in other cases this method exhibited chemoselectivity issues, as diazonium salts can react with a wide variety of amino acid residues, a notable example being histidine cross reactivity.^19^ In order to avoid this issue, Jones *et al.*^20^ decreased the pH to 4.5 and achieved direct PEGylation of salmon calcitonin with a diazonium reagent generated *in situ*, although under these conditions the reaction was slower; after 56 h only 78% conversion was achieved. Furthermore, after it was subsequently purified by ion-exchange FPLC only a modest 24% yield was obtained. The polypeptide also contained histidine and other amino acids with potential cross reactivity (*e.g.* lysine and cysteine) in addition to the single tyrosine, but according to mass spectrometry data only single conjugation was observed even with 20 equivalents of diazonium reagent at pH 4.5. Unfortunately, at pH 5.4 and 7.0 multiple modifications were observed, suggesting that, unlike at lower pH, side reactions with other residues occurred. However, the reaction conditions for optimised selectivity decreased the nucleophilicity of tyrosine as well, thus greatly increasing the reaction time. Under these conditions, 72 h were needed to achieve more than 90% conversion.

The requirement for *in situ* preparation of the reagents or immediately prior to reaction with a given peptide/protein was overcome by Gavrilyuk *et al.*, who first described the bench-stable 4-formylbenzene diazonium hexafluorophosphate reagent.^21^ This stability also allowed for more careful control of the equivalents used, reducing the potential for side reactions. In a test for the selectivity of the reaction on small molecule models of amino acids, tyrosine was found to be modified preferentially, but histidine and to a lesser extent tryptophan and cysteine still exhibited some reactivity. However, the unwanted conversions of these amino acids were only ∼2% at pH 8, leading to the establishment of optimal reaction conditions of pH 8, room temperature and a reaction time of 30 min. Interestingly, another study using a different stable diazonium reagent reported complete selectivity towards tyrosine at physiological pH when modifying the antibody trastuzumab.^22^ Since the development of the first stable diazonium reagent for tyrosine modification, the variety of functional groups that can be introduced with such compounds has been expanded, enabling bioorthogonal click chemistry such as azide–alkyne cycloaddition^23^ or tetrazine-ene reactions.^24^ In addition, the method has been applied to many proteins, including the conjugation of the small-molecule inhibitor aplaviroc to a series of anti-HIV antibodies.^25^

### Diazodicarboxyamides

**Scheme 3:**
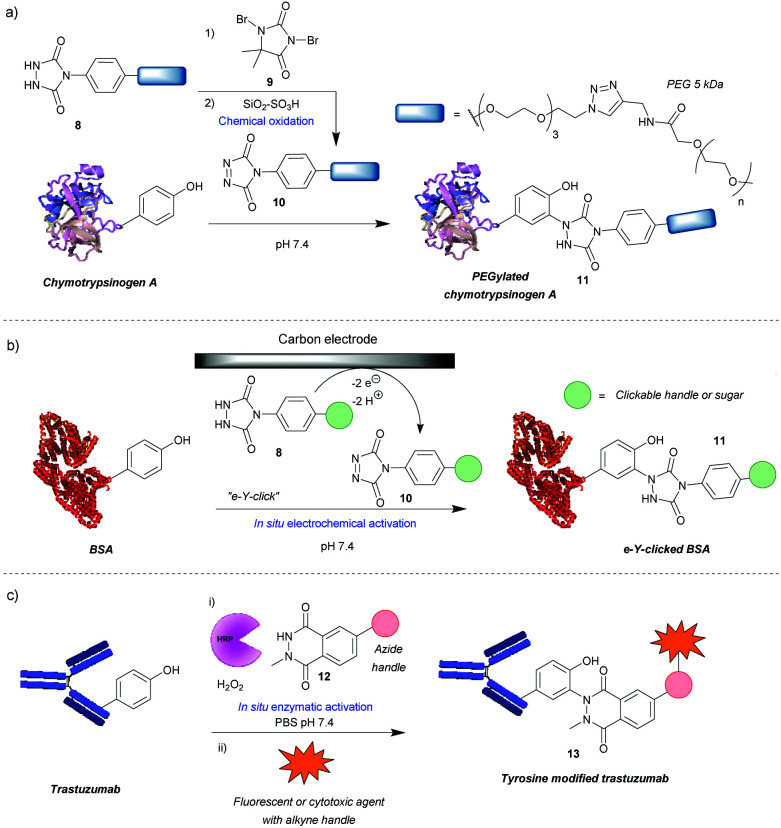
Tyrosine modification strategies based on phenyl-triazolinedione (PTAD) scaffolds. (a) Method relying on chemical generation of active PTAD before reaction. (b) Electrochemical generation of active PTAD *in situ*. (c) Strategy of enzymatically activating a luminol-derivative for tyrosine modification.

One of the most widely used methods for tyrosine modification, first described by Ban *et al.*, utilizes cyclic diazodicarboxyamides, *e.g.* 4-phenyl-3*H*-1,2,4-triazoline-3,5(4*H*)-diones (PTADs) **10** which can be generated from their precursors **8***via* chemical oxidation with reagents such as **9**.^25^,^26^ Compared to previously developed reagents, these compounds react faster, with reaction times typically of 15–30 min, and are selective for tyrosine, unless decomposition takes place (Scheme 3a). For instance, in a peptide containing potentially reactive amino acids, such as lysine, tryptophan and histidine, tyrosine was selectively modified in the presence of a 3-fold excess of PTAD as confirmed by MS/MS data. Products with three different PTADs (all containing electron donating groups, R = OCH_2_C_2_H, OC_2_H_4_N_3_, OCH_2_COCH_3_) were isolated in ∼60% yield.^25^ Another advantage is the stability of the linkage in bioconjugate **11**, which in small molecule models, endures a week-long incubation in human blood plasma, high temperature (120 °C) and pH extremes (10% sodium hydroxide or 10% hydrochloric acid). The reaction can also be conducted over a wide pH range (2–10), although higher yields are generally obtained at higher pHs. In addition, depending on the conditions, the local environment of the tyrosine residue and the chemistry of the PTAD, excellent efficiency can be achieved. For example, a reaction of bovine serum albumin with 5 mM rhodamine-functionalized PTAD (167 equivalents, R = O-PEG_3_-rhodamine) at pH 7.4 resulted in 96% conversion after 15 min^26^ This method proceeded well with electron-donating or neutral substituents present on the phenyl ring, however, it was shown to be inefficient with electron poor PTADs due to their instability. Unfortunately, it was shown that PTADs can decompose to an isocyanate in water, which in turn can cause unwanted side-reactions at lysine residues or the N-terminal amine of the protein. This issue was alleviated by use of 100 mM 2-amino-2-hydroxymethylpropane-1,3-diol (Tris) buffer, which acts as an isocyanate scavenger, thus reducing side reactions.^27^ Despite this limitation, a variety of PTADs were synthesised, some bearing functionality for subsequent click reactions. Furthermore, the method has been successfully applied in a wide array of protein modifications reactions, including: attachment of fluorescent markers to proteins, PEGylation, preparation of antibodies with multiple selectivity, antibody–drug conjugates (ADCs), glucoconjugate vaccines^27^ and protein–DNA conjugates.^28^ Although not exclusively, in many of these cases, predominantly monoconjugation was observed. In addition, this method is orthogonal to lysine and cysteine modification, which allows for dual- or even triple-modification, as shown by the sequential conjugation of bovine serum albumin with 11-(dansylamino)undecanoic acid, a PTAD derivative and fluorescein-5-maleimide.^25^

Recently, an alternative method, employing electrochemistry to generate PTADs **10** from 4-phenylurazole precursors **8***in situ*, was developed; termed the electrochemical tyrosine click (e-Y-click).^29^ The applied potential was low enough to not oxidise amino acid residues nor the product of conjugation (Scheme 3b). Most importantly, PTAD decomposition and side reactions of the by-product with lysine were observed to be far reduced, therefore eliminating the need for Tris buffer as a by-product scavenger, allowing a more flexible choice in deciding what buffer and pH to use. This approach proved to be more efficient than using chemical oxidation to synthesise PTADs, as under similar conditions and reagent equivalents (30 eq.), it resulted in the labelling of more than twice as many tyrosine residues on bovine serum albumin (average of 9.1 as opposed to 3.7). This pioneering work also inspired further research in the field of electrochemically promoted tyrosine-selective protein labeling.^30^,^31^

Another way to avoid side reactions was to use luminol derivatives **12** that do not decompose to isocyanates.^32^*N*-Methyl luminol derivatives activated *in situ* with hydrogen peroxide and hemin as a catalyst were conjugated to angiotensin II with 95% conversion (Scheme 3c). The modification was predominantly selective for tyrosine and no electrophilic by-products were formed as determined by MS/MS. In contrast, PTAD was shown to modify a lysine residue as well as the N-terminal amine after decomposition to phenylisocyanate, even in the presence of 100 mM Tris buffer. However, the required large excess of hydrogen peroxide led to oxidation of a significant proportion of the cysteine residues over the course of the reaction, 40% in the case of BSA as judged by the inability to modify them with maleimide post-tyrosine modification. This side reaction was minimised by using a better catalyst – horseradish peroxidase (HRP), which enabled the use of lower amounts of hydrogen peroxide or β-nicotinamide adenine dinucleotide (NADH) instead of hydrogen peroxide,^33^ or by using atmospheric oxygen dissolved in the solution with the catalyst laccase.^34^ The laccase method was shown to be more effective than that relying on HRP and hydrogen peroxide or the electrochemical reaction with PTAD (e-Y-click). However, it did oxidize cysteines (38%) more than the e-Y-click (15%), and only slightly less than the HRP method under these conditions (49%). Sato *et al.* not only demonstrated that the luminol derivatives are more efficient for tyrosine modification than the PTADs under these conditions, but also are better substrates for the e-Y-click.^31^ In addition, they succeeded in site-selective modification of several proteins owing to the unique high solvent accessibility of one or more tyrosine residues, which further reinforces the robustness of tyrosine modification in particular cases.^31^ For example, streptavidin was selectively modified on Y83, trastuzumab on Y57 and rituximab on all four of its solvent accessible tyrosine residues. The antibodies were modified in the complementarity-defining regions, where the tyrosine residues are exposed in contrast to the buried ones in the constant regions. As expected, this reduced their antigen-binding affinity (*e.g.* 7-fold in the case of trastuzumab after modification and 20-fold after subsequent click of fluorophore). Nevertheless, the trastuzumab ADC demonstrated selective cytotoxicity, and therefore this method could be useful in cases where a higher *K*_D_ is beneficial. Lower monovalent binding could improve selectivity for over-expressing cell populations.^35^ Alternatively this method could find use in chemically tuning the affinity of antibodies, say in the case of bispecifics,^36^ where a *K*_D_ mismatch between paratopes may be disadvantageous.

### Transition metal-mediated approaches

**Scheme 4:**
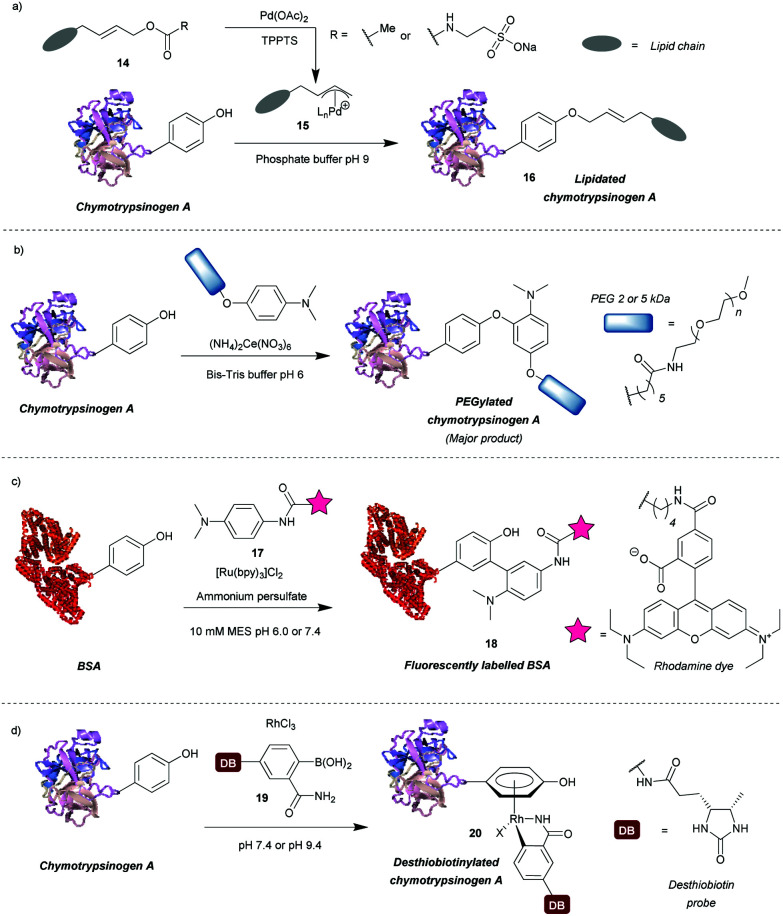
Transition metal-based approaches for tyrosine modification. (a) An electrophilic π-allyl palladium complex for tyrosine *O*-alkylation. (b) A method relying on a cerium(iv) salt to generate tyrosyl radicals. (c) A ruthenium(ii) photocatalyst to generate tyrosyl radicals for subsequent modification. (d) Bioconjugation of tyrosine residues with an η^6^ Rh(iii) complex.

A number of transition metal-mediated approaches are available for tyrosine modification, from their use as oxidants or catalysts to organometallic bioconjugation. Tilley *et al.*^37^ explored the reaction of tyrosine with an electrophilic π-allyl palladium complex **15**, which results in tyrosine *O*-alkylation. The complex is generated from allylic acetate **14** in the presence of palladium(ii) acetate (Scheme 4a). Chymotrypsinogen was modified with 50–65% conversion (monoadduct) using this procedure at pH 8.5–9 in 45 min. The reaction was selective for tyrosine as determined *via* proteolytic digestion and MS analysis of the fragments. Moreover, proteins with no surface accessible tyrosines, but with exposed lysines (horse heart myoglobin) and cysteines (H-Ras) were not modified under the reaction conditions. The method enabled attachment of particularly hydrophobic moieties (*e.g.* a farnesyl group) in order to produce proteins with the ability to incorporate into lipid bilayers. The initial compound contained the hydrophobic moiety but this was solubilised by a very hydrophilic group (*e.g.* taurine carbamate) that was attached in place of acetate. This hydrophilic group (just like the acetate) was removed on formation of the π-allyl Pd complex, and was thus not attached to the final conjugate. Farnesylated chymotrypsinogen A was generated using this method and shown to be incorporated into a lipid bilayer.

Metal ions can also participate in single electron transfer reactions on tyrosine residues. Seim *et al.*^38^ described the oxidative coupling of substituted anilines with tyrosine in the presence of cerium(iv) ammonium nitrate as an oxidant. The reaction yields predominantly *O*-alkylated product with some *C*-alkylation also observed (ratio 85 : 15). Using this method chymotrypsinogen was conjugated with PEGs (2–5 kDa) and conversions between 45% and 71% were achieved in 1 h. Depending on the reagent, the reaction can also modify tryptophan, however, it was shown that anisidine-based compounds are selective for tyrosine. The reaction also leads to cysteine oxidation but it was shown that dual-modification can be attained by labelling the cysteine residues with a maleimide-fluorophore before tyrosine-modification. Alternatively, tyrosyl radicals can be generated in the presence of photocatalysts such as ruthenium(ii) tris(2,2′-bipyridyl) complex ([Ru(bpy)_3_]^2+^).^39^ This species was subsequently reacted with tyrosyl radical trapping agents, *e.g.* electron-rich anilines **17**^39^ or PTAD precursors – 1-methyl-4-aryl-urazoles (MAUras).^40^ Angiotensin II was modified with up to 95% conversion (combined for mono- and diadduct, products of *C*-arylation) at pH 7.4 after only 1 min of light irradiation (Scheme 4b).^39^ In addition, bovine serum albumin (BSA) and streptavidin were successfully fluorescently labelled and subsequent conjugation with maleimide was also successful, suggesting that cysteine residues were not oxidised in the process. This method has been mainly applied as a tool to selectively label target proteins in the presence of other proteins using ligand-conjugated photocatalysts.^40^

Recently, Ohata *et al.*^41^ developed an alternative organometallic bioconjugation method, which involved formation of an η^6^ Rh(iii) – tyrosine complex **20** (Scheme 4c). Functionality or cargo were introduced *via* transmetalation of arylboronic acids **19** bearing an *o*-carboxamide substituent necessary for efficient reaction. Interestingly, no reaction was observed with *p*-cresol under the same conditions peptides were modified. The authors suggest that perhaps peptide structure is required for successful reaction, but further investigation of this phenomenon would be beneficial. Angiotensin IV was conjugated with (2-carbamoylphenyl)boronic acid overnight at pH 9.4 with 92% conversion. In addition, the antibody Herceptin™ and other proteins (*e.g.* chymotrypsinogen, ovalbumin, BSA) were conjugated with a fluorescent dye or desthiobiotin, with the antibody retaining antigen-binding ability. It is worth mentioning that although the linkage is stable to plasma thiols, *e.g.* glutathione, the labelling is diminished upon addition of dithiothreitol or hydrogen peroxide, which as suggested by the authors may find applications in controlled cargo release.

### Methods based on sulfur-fluoride or triazole exchange

**Scheme 5:**
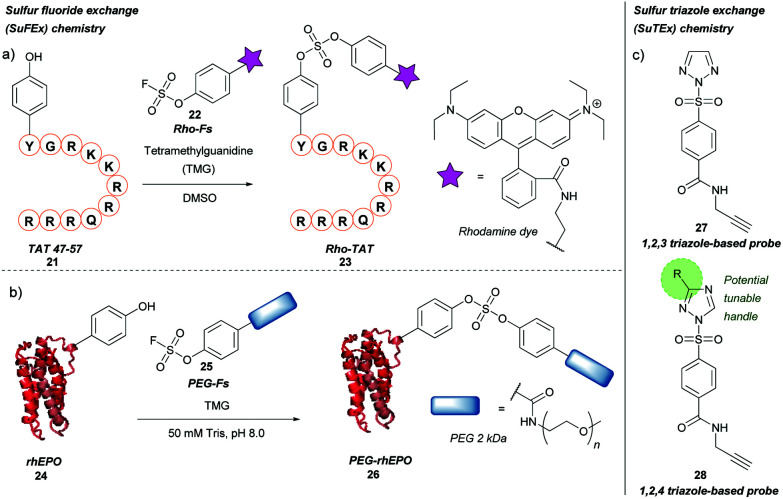
Sulfur fluoride or triazole exchange strategies for tyrosine bioconjugation. (a) Sulfur fluoride exchange (SuFEx) chemistry. (b) Sulfur triazole exchange (SuTEx) chemistry.

Previously described for its potential in click chemistry^42^ and use in probing tyrosine residues in cell proteome analysis,^43^ sulfur fluoride exchange (SuFEx) chemistry has recently been used in the context of tyrosine bioconjugation (Scheme 5a).^44^ Initially, the SuFEx bioconjugation was validated through reaction of *p*-cresol with phenyl fosylate and different bases; of the bases screened, addition of 1,8-diazabicycloundec-7-ene (DBU) or tetramethylguanidine (TMG) gave quantitative conversion to the desired sulfate. The reactivity of phenyl fosylate with other potential amino acid residues was next established *via* screening of a panel of small molecule model nucleophiles, as reactive side-chain analogues, against phenyl fosylate in the presence of TMG in DMSO. Propanethiol (Cys), methanol (Ser), *N*-propylguanidine (Arg), and *n*-butylamine (Lys) led to no expected product formation after 12 h of reaction time. However, a 12.5% isolated yield of product was observed when 3-methylindole (Trp) was used as a nucleophile. Reaction with 4-methylimidazole (His) was almost completely prevented through addition of Ni^2+^. In contrast, a 93% isolated yield of product was obtained within 90 min when *p*-cresol (Tyr) was used as the nucleophile of choice. SuFEx tyrosine bioconjugation was further validated through modification of TAT 47–57 **21**, a cell penetrating peptide (CPP) fragment containing a single tyrosine residue at the N-terminus. Reaction of TAT 47–57 with rhodamine-conjugated aryl fosylate (Rho-Fs, **22**) in the presence of TMG in DMSO lead to complete conversion to the fluorescently labelled peptide (Rho-TAT, **23**) as judged by MALDI-TOF mass spectrometry. Selectivity for the tyrosine residue was confirmed by MALDI-TOF/TOF tandem mass spectrometry. Despite the presence of nearby arginine residues, which had previously been postulated to facilitate tyrosine modification of proteins *via* SuFEx, no product formation occurred unless TMG was added to the reaction; this was hypothesised to be due to differing peptide/protein microenvironments compared to previous reports. Rho-TAT demonstrated cell permeability in HeLa cells and could be used to visualise cell nuclei *via* fluorescent imaging. SuFEx tagging was then applied towards protein bioconjugation under aqueous conditions. For this, recombinant human erythropoietin (rhEPO, **24**), which contains a single surface-exposed tyrosine (Tyr49) anticipated to be a suitable bioconjugation site, was chosen. Reaction of rhEPO with fosylated polyethylene glycol **25** (*M*_n_ ≈ 2000) in Tris buffer (50 mM, pH 8.0) with TMG gave the PEGylated rhEPO (PEG-rhEPO, **26**) as confirmed by MALDI-TOF after a 3 h reaction time. A mass shift of ∼2000 Da was observed, suggesting a high degree of modification. Selective modification at Tyr49 was further confirmed by trypsin digestion and MALDI-TOF experiments, with no modification of internal tyrosine residues or histidine residues observed. PEG-rhEPO **26** was subsequently shown to retain its function *in vivo* (*i.e.* induction of red blood cell production) post SuFEx-mediated PEGylation.^44^

More recently, a related strategy for tyrosine labelling that utilises sulfur-triazole exchange (SuTEx) chemistry has been reported for probing of tyrosine residues in human cell proteomes, including ‘hyper-reactive’ tyrosine residues, and sites of tyrosine phosphorylation (Scheme 5b).^45^ Here, the fluorine leaving group (which is required for SuFEx activation) was replaced with either a 1,2,3 or 1,2,4 triazole group (compounds **27** and **28**, respectively); unlike fluorine, the triazole motifs have the potential to be functionalised further, which would allow for tuning the properties of a given chemical probe (*e.g.* reactivity, selectivity). Triazole-bearing SuTEx probes containing a clickable handle showed greater reactivity and higher specificity towards tyrosine over lysine compared to fluorine-containing SuFEx probe analogues when used in proteomics experiments; for example, *p*-methoxy substituted 1,2,4 triazole SuTEx probe showed improved tyrosine selectivity (Tyr/Lys selectivity ratio ≈ 5/1) compared to fluorine-containing SuFEx probe (Tyr/Lys selectivity ratio ≈ 2.3/1). This method was used to investigate tyrosine phosphorylation of the proteome, as phosphorylation of Y residues was shown to compete with SuTEx labelling. This Y/K ratio was later improved to ≈10/1 by further optimization of the adduct and leaving groups.^46^

It would be interesting to see how this method could be adapted to tyrosine bioconjugation chemistry, and whether the modest selectivity for Y over K observed in these proteomics experiments could be improved by further tuning the triazole substituents. In any case, it will be worth following the developments in SuTEx chemistry for those interested in the synthesis of useful and homogenous protein bioconjugates.^45^

### Chemical *O*-glycosylation of tyrosine

**Scheme 6:**
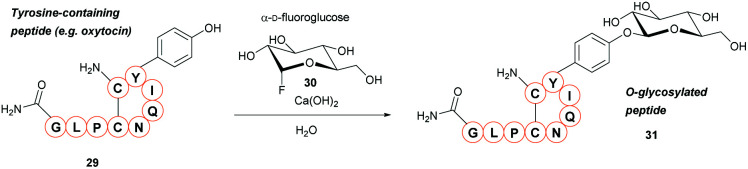
Glycosylation of tyrosine residues with α-d-fluoroglycosides.

A new method for synthetic *O*-glycosylation of tyrosine containing peptides using fluoroglycosyl donors has very recently been described (Scheme 6).^47^ In contrast to previously reported methodology, phenolic *O*-glycosylation *via* this strategy could be performed in a protecting-group-free manner under aqueous conditions. To begin with, *O*-glycosylation of Boc-l-Tyr-OH with α-d-fluoroglucose **30** was performed in aqueous conditions in the presence of various additives. Of the additives screened, addition of Ca(OH)_2_ gave the desired *O*-glycosylated product **31** in a 94% conversion (NMR) within 10 min. Changing Ca(OH)_2_ for Ca(OTf)_2_, or using other alkaline earth metal hydroxides (*e.g.* LiOH, Mg(OH)_2_, Ba(OH)_2_ as the additive of choice gave significantly lower conversions (0–8%, as judged by NMR). Only β-glucosyl products were observed, with no epimerisation at the anomeric position even after 24 h of reaction time. Other α-d-fluoroglycosides, including α-d-fluorogalactose and α-d-fluoromaltose could also be used, resulting in the respective β-glycosides. In contrast, when α-d-fluoromannose was used, a significantly lower yield (isolated) and the α-anomer as the major product was obtained; furthermore, attempts to react β-d-fluoroglucose with 4-methoxyphenol led to the β-anomer glycoside. The α-d-fluoroglycosyl donors also react with free cysteine; reaction of Boc-Cys-OH leads to the *S*-glyosylation product in 98% yield (isolated), whereas a mixture of *O*-glysolated and *S*-glycosylated products are observed when using Boc-Tyr-Cys-OH. Aside from this, the methodology showed good functional group tolerance, and was applied to rapid tyrosine *O*-glycosylation of biologically active peptides, including glucagon-like peptide 1 (46% conversion), vasopressin (67% conversion), oxytocin (68% conversion), and calcitonin (49% conversion). In all cases some off-target glycosylation was observed, but due to the small amounts of these by-products, MS/MS analysis was unable to confirm their exact identities. It is important to note that none of these peptides contained free cysteine side-chains. Indeed, on a model dipeptide, Boc-Tyr-Cys-OH, the cysteine side-chain was glycosylated to a greater extent (69% as opposed to 46%).^47^

## Chemoenzymatic approaches for tyrosine modification

### Enzyme-mediated modifications

**Scheme 7:**
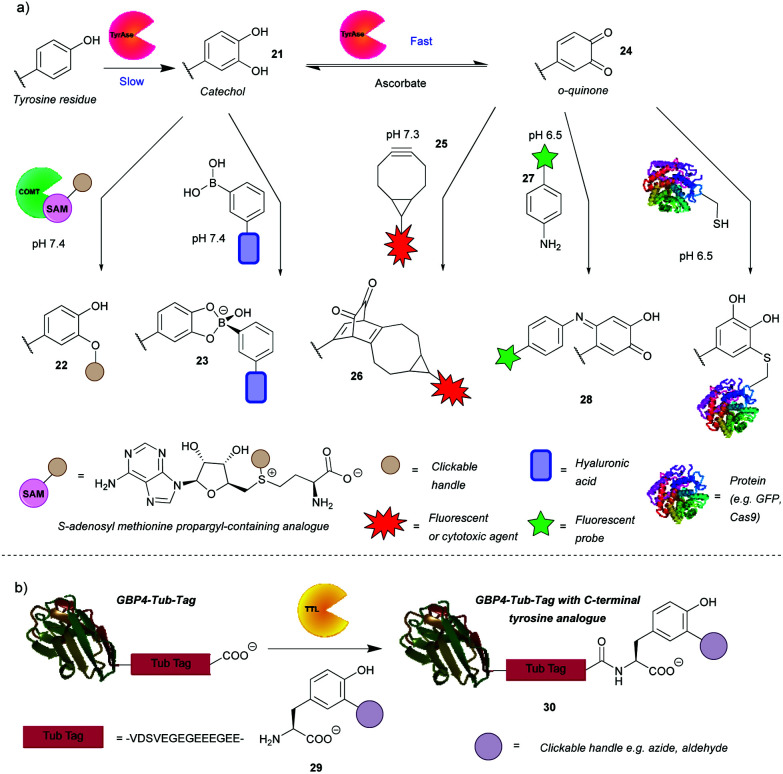
Enzyme-mediated strategies for tyrosine bioconjugation. (a) Strategies relying on the enzyme tyrosinase. The catechol generated *via* this enzyme can be *O*-alkylated or reacted with boronate esters. If oxidation is allowed to progress to the *o*-quinones, these can be reacted with bicyclononynes in a strain-promoted cycloaddition or with N or S nucleophiles. (b) Strategy relying on incorporation of unnatural tyrosine residues *via* the enzyme tubulin tyrosine ligase.

In addition to the direct chemical modification of tyrosine, strategies involving enzymatic transformation have also been developed. Many enzymes oxidise tyrosine, including the aforementioned HRP. Besides activating luminol derivatives, it can generate tyrosine radicals, which can in turn, for example, form dityrosine, crosslinking peptides or react with other species generated by HRP such as tyramide radicals. This topic and its applications, *e.g.* peroxidase-proximity protein labeling, were recently reviewed by Sato and Nakamura.^48^ In view of this, this review will first focus on another enzyme gaining attention in the field – tyrosinase. Unfortunately, it is difficult to access even surface-exposed tyrosine residues on many proteins when using this enzyme, due to its steric bulk. As such, it often requires a tyrosine-containing linker, such as the hemagglutinin (HA) tag or the tyrosine tag (-G_4_Y), that extends far enough into the solvent to enable modification. This is evident from the numerous examples of tagged proteins (*e.g.* End35, laminarinase A, trastuzumab fragment) being successfully modified by tyrosinase, with no reaction occurring with the wild type under the same reaction conditions (Scheme 7a).^49^–^51^ Although having to genetically modify the protein is generally a downside (*e.g.* increased cost, time *etc*.), it does ensure site-selectivity.

Tyrosinase catalyses the oxidation of tyrosine to DOPA **32** in the first slower step and then the oxidation of DOPA to *o*-quinone **35**, in a subsequent faster step. It is therefore challenging to obtain the catechol product. However, Struck *et al.*^49^ and Montanari *et al.*^52^ both succeeded in this endeavour by adding ascorbic acid to the reaction mixture to reduce the *o*-quinone **35** back to the catechol **32**. Montanari *et al.*^52^ then chemically conjugated DOPA to functionalised boronic acids, which was used to link ovalbumin with hyaluronic acid. According to the calculated equilibrium constants, the authors concluded that the boronic esters are obtained from catechols quantitatively, however, an important thing to note is that the linkage is not particularly stable as the esters hydrolyse at mildly acidic pH (5–6.5). This is a drawback for general applications, but it could be exploited for specific applications where pH-triggered release is advantageous, such as intracellular delivery followed by lysosomal release of cargo. Alternatively, Struck *et al.*^49^ used another enzyme, catechol *O*-methyl transferase (COMT), to *O*-alkylate DOPA to form bioconjugates such as **33**. In addition to transferring a methyl group, COMT is able to introduce more useful functionalities, *e.g.* a propargyloxybut-2-enyl group, which can participate in a subsequent azide–alkyne cycloaddition. The two enzymes can be combined in a one-pot reaction, thus shortening the procedure. Applying this to a peptide with an N-terminal tyrosine resulted in 76% conversion after 5 h. Although proteins, engineered to have N-or C-terminal tyrosine, were methoxylated, attachment of cargo is yet to be demonstrated on systems that are more complex than peptides.

The *o*-quinones (*e.g.* compound **35**) produced in the second step can be attacked by nucleophiles, which is one approach to enable their modification.^53^ The nucleophiles need to be reactive enough to compete with side reactions such as hemagglutinin tag cleavage or *o*-quinine polymerization^53^ or attack from the side chains of lysine, histidine and/or cysteine resulting in crosslinking.^54^ As a proof of concept, Long and Hedstrom used Cy5-hydrazide to label proteins, *e.g.* dihydrofolate reductase.^53^ Recently, the suitability of anilines and cyclic amines as nucleophiles was compared, with anilines (*e.g.* compound **28**) exhibiting higher efficiency.^51^ Therefore, cargo-functionalised anilines were used for tyrosinase activated bioconjugation to a peptide and an antibody fragment. To illustrate the method, a single chain variable fragment (scFv) of trastuzumab bearing a -GGY tag was labelled site-specifically with the fluorescent dye Oregon Green in 1 h with 84% conversion. Antigen recognition was preserved post-conjugation as proven by the selectivity for HER2(+) cancer cells in a flow cytometry experiment. A disadvantage of this reaction, however, was that the generated *p*-iminoquinones (such as **39**) formed adducts with glutathione and DTT. That being said, glutathione adducts were formed quantitatively over 24 h and without detachment of the cargo. Additionally, the glutathione adduct was stable to addition of DDT; no competitive release was observed. Thus, pre-treatment with glutathione might avoid problematic thiol conjugation in blood. It was also found that the initially used tyrosinase from the button mushroom *Agaricus bisporus* (abTYR) was unable to oxidize protein L constructs prepared with tyrosine-containing tags. This issue was postulated to be caused by steric hindrance of the bulky 120 kDa enzyme, and indeed was circumvented by using the more compact 35.5 kDa *Bacillus megaterium* tyrosinase (bmTYR).

Although sometimes a reason for side reactions, the attack of nucleophilic amino acids on *o*-quinones can be exploited to prepare site-specific protein-peptide or protein–protein conjugates. This was demonstrated recently by Lobba *et al.*^55^ with the addition of surface-exposed cysteine thiols of one protein to *o*-quinones obtained by tyrosinase catalysed oxidation of tyrosine on another protein or peptide. The attack occurs at the 5-position of the *o*-quinone (numbered from the amino acid branching point) and the resulting product is predominantly in its catechol form. This is in contrast to the aniline nucleophiles, which add to the 6-position and the products of which exist mainly in quinone form.^51^ The cysteine/*o*-quinone linkage formed was shown to be more stable compared to an analogous thiosuccinimide linkage after incubation in human blood serum for 7 days.^55^ The method was used to attach a variety of proteins and peptides to other proteins. For example, GFP bearing a tyrosine tag was conjugated successfully to Cas9. A HER2-binding scFv with tyrosine tags was also conjugated to mutant GFP with an introduced cysteine residue. Quantitative conversion was achieved in both cases. The modified Cas9 retained its DNA cleavage ability, and the antigen binding affinity of the scFv was preserved. These reactions proceeded under mild conditions (4 °C or room temperature, pH 6.5 or pH 7.0) and with short reaction times usually between 30 min and 1 h. It is worth noting that the oxidized N-terminal tyrosine residues can undergo dopachrome-like cyclization. In order to prevent this, N-terminal acylation or addition of an N-terminal glycine was carried out.

Enzymatically generated *o*-quinones can also participate in a strain-promoted cycloaddition with bicyclo[6.1.0]nonyne (BCN) derivatives (*e.g.* compound **36**).^50^ Site-specific modifications of tyrosine tagged proteins, including antibody–drug conjugates, were obtained in 30 min. Interestingly, while the reaction was successful over a range of temperatures (4, 16 and 37 °C and room temperature) with laminarinase A (LamA), trastuzumab could only be modified at 4 °C and 16 °C; no conjugated product was detected at 37 °C. As suggested by the authors, this is probably due to an intramolecular reaction with a nearby nucleophilic amino acid residue. Additionally, an anti-influenza antibody, AT1002 equipped with a Y-tag was also converted to the corresponding ADC. Conversion of LamA was observed to be ∼80% by MS, while conversion of AT1002 light chain was shown to be in the 40–70% range. Even so, AT1002 was shown to be labelled more efficiently than trastuzumab, possibly due to the longer spacer between the protein and the Y-tag allowing easier access for the bulky enzyme. It would be interesting to see if a smaller tyrosinase would offer increased conversion in this case as well. Although there is room for optimisation, this is a promising method for protein conjugation.

Enzymes can not only modify existing tyrosine residues, but also install new ones onto the protein of interest for subsequent modification. Tubulin tyrosine ligase (TTL) recognises a short sequence on the C-terminus of tubulin (“Tub-tag”) and attaches a tyrosine (Scheme 7b). This is particularly useful, because the enzyme is flexible and can work with unnatural tyrosine derivatives (*e.g.* reagent **40**), with additional functionality for click reactions, *e.g.* 3-azido- and 3-formyltyrosine,^56^ which are otherwise difficult to incorporate during synthesis and often lead to low protein yield. Schumacher *et al.* fused tub-tag onto green fluorescent protein (GFP), ubiquitin and GFP single-domain nanobodies and attached a variety of tyrosine derivatives using this method. Subsequent click reactions enabled conjugation with biotin, fluorophores and PEG. As an example of the reaction efficiency, at a TTL/GBP4 ratio of 1 : 10, 82% and 99% of the GBP4 nanobody equipped with a Tub-tag had 3-azidotyrosine attached after 1 and 3 h, respectively, at 37 °C. Unsurprisingly, the following conjugation *via* click-chemistry was site-specific and efficient. The method was also used to label GFP with biotin selectively in cell lysate, which was then purified in a pull-down assay. Fluorophore labelled nanobodies were also used in super-resolution microscopy experiments.

### Ribozyme-catalysed modifications

**Scheme 8:**
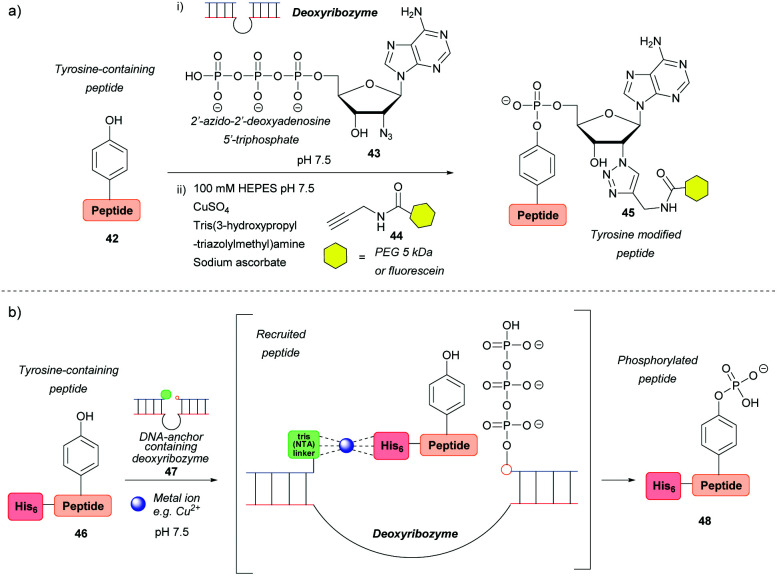
Deoxyribozyme-catalysed tyrosine modification. (a) Functionalising an untethered tyrosine-containing peptide *via* deoxyribozyme-mediated azido-adenylylation. (b) Non-covalent recruitment and phosphorylation of an untethered peptide *via* metal chelation to a deoxyribozyme.

Deoxyribozymes can catalyse the nucleophilic attack of tyrosine on a triphosphate and they have been exploited to facilitate the ligation of tyrosine with a 5′-triphosphorylated RNA^57^,^58^ and 2′-azido-2′-deoxyadenosine 5′-triphosphate.^59^ The reaction results in an RNA–peptide conjugate or introduction of an azide group to enable further modification (Scheme 8a). Although high selectivity was achieved, with some deoxyribozymes being able to discriminate between two tyrosine residues on a single peptide, a lengthy selection process is needed in order to find an efficient catalyst in the first place, which makes the method inefficient in terms of time and cost. In addition, the method works best when the substrate is tethered to the deoxyribozyme through a complementary DNA anchor. Without this tethering, which necessitates pre-modification, 14% and 59% azide-functionalization were achieved for the 32-mer salmon calcitonin (sCT) and a 28-mer fragment of atrial natriuretic peptide (atriopeptin, ANP), respectively, over 24 h.^59^ Both azido-peptides were successfully modified further using CuAAC. A slightly different approach used a hexahistidine tag (His_6_) on the untethered peptide **46** in combination with a DNA anchor complementary to part of the deoxyribozyme and linked to three nitrilotriacetic acid (NTA) units **47**. This arrangement would bring the substrate and the catalyst into close proximity^60^*via* binding of divalent metal ions (*e.g.* Cu^2+^) to both the His_6_ and NTA (Scheme 8b). The strategy increased the conjugation efficiency more than 6-fold relative to a control without this recruitment. But even so, conversion was only 44% for the 24-mer peptide tested. With respect to protein modification, this procedure was unsuccessful as no conversion was observed when tested on three different proteins (the natively metal-binding carboxypeptidase B and His_5_ tagged lysozyme and plasminogen activator inhibitor **1**). The authors attributed this to the deoxyribozyme not having access to peptide sequences in the protein structure.

## Conclusions

To conclude, due to its relatively low abundance on the protein surface, tyrosine is a promising target for site-selective bioconjugation of naturally occurring proteins. In the case of some proteins, there are a few or only one particularly solvent-accessible tyrosine residue, which allows chemical approaches to achieve highly homogenous products. Examples include the TMV capsid modified on Y139 with diazonium reagents^18^ as well as streptavidin (Y83) and trastuzumab (Y57) conjugated with diazodicarboxyamides.^31^ Alternatively, site-selectivity can be achieved by genetically engineering the protein to express a single accessible tyrosine residue. Although such methods are less time- and cost- effective as a whole due to the preceding preparatory steps, the tyrosinase-catalysed modification of tyrosine-containing tags and subsequent chemical conjugations proceeds quickly and with high conversions under physiological conditions.^49^–^52^ The ligation of unnatural tyrosine derivatives to proteins has been also successfully achieved.^56^ In addition, tyrosine tags are typically small and loss of protein activity was not reported in any of the examples reviewed.

However, overall, there are as of yet only a handful of methods for tyrosine bioconjugation developed. Nonetheless, the recent developments in the field demonstrate the opportunities that tyrosine modification opens up and its potential to complement the more traditional cysteine and lysine modification methods. For instance, should a protein contain both a single cysteine and tyrosine residue, site-selective dual modification could be achieved. While the field is certainly in a less mature phase of development in comparison to other amino acid modification fields, this constitutes an opportunity, as with further work the current scope and potential it provides could be expanded prodigiously. Should tyrosine modification attain the level of maturity and efficacy that the more common strategies have, it could offer some advantages, *e.g.* the possibility of site-selective modification due to low natural abundance. Thus, we believe there certainly is much work to be done in the field, as well as good reason to do so.

## Conflicts of interest

V.C. is a co-founder and director of the company ThioLogics.
