# Correction: Tyrosine bioconjugation – an emergent alternative

**DOI:** 10.1039/d2ob90141b

**Published:** 2022-10-31

**Authors:** Peter A. Szijj, Kristina A. Kostadinova, Richard J. Spears, Vijay Chudasama

**Affiliations:** Department of Chemistry, University College London London UK v.chudasama@ucl.ac.uk

## Abstract

Correction for ‘Tyrosine bioconjugation – an emergent alternative’ by Peter A. Szijj *et al.*, *Org. Biomol. Chem.*, 2020, **18**, 9018–9028, https://doi.org/10.1039/D0OB01912G.

The authors regret that there were some errors in the compound numbering shown in Scheme 7. The correct scheme is shown below.

**Scheme 1:**
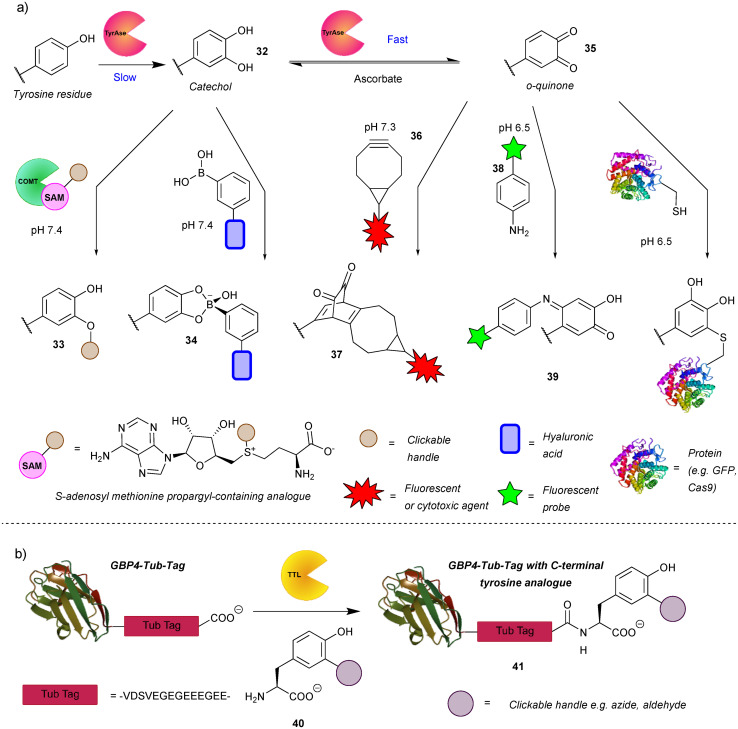
Enzyme-mediated strategies for tyrosine bioconjugation. (a) Strategies relying on the enzyme tyrosinase. The catechol generated *via* this enzyme can be *O*-alkylated or reacted with boronate esters. If oxidation is allowed to progress to the *o*-quinones, these can be reacted with bicyclononynes in a strain-promoted cycloaddition or with N or S nucleophiles. (b) Strategy relying on incorporation of unnatural tyrosine residues *via* the enzyme tubulin tyrosine ligase.

In addition, in the paragraph starting ‘The *o*-quinones (*e.g.* compound **35**) produced in the second step can be attacked by nucleophiles’, the sentence ‘Recently, the suitability of anilines and cyclic amines as nucleophiles was compared, with anilines (*e.g.* compound **28**) exhibiting higher efficiency.^51^’ should read ‘Recently, the suitability of anilines and cyclic amines as nucleophiles was compared, with anilines (*e.g.* compound **38**) exhibiting higher efficiency.^51^’

The Royal Society of Chemistry apologises for these errors and any consequent inconvenience to authors and readers.

## Supplementary Material

